# Differential Diagnosis of Multiple System Atrophy-Parkinsonism and Parkinson's Disease Using α-Synuclein and External Anal Sphincter Electromyography

**DOI:** 10.3389/fneur.2020.01043

**Published:** 2020-09-17

**Authors:** Zhentang Cao, Yufeng Wu, Genliang Liu, Ying Jiang, Xuemei Wang, Zhan Wang, Tao Feng

**Affiliations:** ^1^Department of Movement Disorders, Center for Neurology, Beijing Tiantan Hospital, Capital Medical University, Beijing, China; ^2^China National Clinical Research Center for Neurological Diseases, Beijing, China; ^3^Department of Laboratory Medicine, Peking University Third Hospital, Beijing, China; ^4^Parkinson's Disease Center, Beijing Institute for Brain Disorders, Capital Medical University, Beijing, China

**Keywords:** multiple system atrophy, Parkinson's disease, α-synuclein, external anal sphincter electromyography, saliva

## Abstract

**Background and aim:** Discriminating multiple system atrophy-parkinsonism (MSA-P) from Parkinson's disease (PD) is challenging. We aimed to provide a new method to make an identification between MSA-P and PD by combining biofluid marker with electrophysiology marker.

**Methods:** The XYCQ EV Enrichment KIT was applied to extract extracellular vesicles (EVs) from saliva. The levels of α-syn which included total α-syn (α- syn_Total_), phosphorylated-ser129 α-syn (α-syn_PS129_) and oligomeric α-syn (α-syn_Olig_) in EVs of saliva were tested by new developed Electrochemiluminescence (ECL) assays. We collected multi-motor unit potential (MUP) of all participants who conducted external anal sphincter electromyography (EAS-EMG). The duration, phase, amplitude and satellite potential of EAS-EMG were analyzed. The Receiver operator characteristic (ROC) curve was adopted to analyze the diagnostic utility of α-syn in EVs of saliva, EAS-EMG for MSA-P.

**Results:** In EVs of saliva, the α-syn_Total_ concentrations were lower in MSA-P than PD (*P* = 0.003). No significant difference was shown in α-syn_Olig_ and α-syn_PS129_. α-syn_Total_ 4.46 pg/ng distinguished MSA-P from PD with area under the curve (AUC) 0.804. Compared with PD, the duration, phase and satellite potential of EAS-EMG in MSA-P were increased (*P* = 0.002, 0.008, 0.001). There was no significant difference in amplitude. ROC curve showed that the duration (AUC: 0.780), phase (AUC: 0.751), and satellite potential (AUC: 0.809) had both diagnostic value for MSA-P. The combination of α-syn_Total_ in salivary EVs and EAS-EMG (including duration, phase and satellite potential) could efficiently make a differentiation between MSA-P and PD with sensitivity of 100% and specificity of 86%. The AUC value was 0.901.

**Conclusion:** The study suggested the combination of α-syn_Total_ in salivary EVs and EAS-EMG could help efficiently distinguish MSA-P from PD.

## Introduction

Synucleinopathy is one kind of neurodegenerative disease featured by the aggregation of insoluble α-synuclein (α-syn). These pathological inclusions often deposited in the neurons or in the glia cell (1, 2). Multiple system atrophy (MSA) is classified as α-synucleinopathy. The main clinical characteristics of MSA included cerebellar ataxia, disorder of autonomic nerve function, poorly levodopa-responsive parkinsonism, and pyramidal symptom (3). The two phenotypes of MSA were Parkinsonian (MSA-P) and cerebellar entity (MSA-C). The diagnosis of MSA is principally depended on clinical symptoms, with a high risk of misdiagnosis. Especially MSA-P shares similar neuropathological, cognitive and clinical profiles with PD, complicating its diagnosis. In consequence, it is robustly urgent to recognize significant markers which could effectively distinguish MSA-P and PD.

α-syn is the main component of intracellular depositions that detected in the Lewy bodies (LBs) of PD or glial cytoplasmic of MSA, which is widely expressed in the brain (4). It is also aggregated in the extracellular biofluids just as blood, cerebrospinal fluid (CSF), and plasma (5–7). Meanwhile, several studies have shown that α-syn was promising to become diagnostic biomarker because the levels of α-syn in CSF are related to synucleinopathy (7). In CSF, Shi et al. (8) found that α-syn levels in CSF were decreased regardless of PD and MSA. In plasma (6), the α-syn levels were increased in PD and MSA patients. In red blood cells (9), no statistical difference was shown in the ratio of oligomeric α-syn/total protein. Therefore, only measurement of α-syn may be insufficient for MSA differential diagnosis especially for MSA-P. And recently the study showed that α-syn originating from plasma extracellular vesicles (EVs) was a promising biomarker for the diagnosis of PD (10). But the difference of α-syn in EVs of saliva in MSA and PD was unknown.

The prominent clinical symptom of MSA with the neurodegeneration of Onuf's nucleus (11, 12) is autonomic dysfunction. Neurogenic damage detected by EAS-EMG showed either Onuf's nucleus involvement, or pudendal nerve damage. Nonetheless, PD can also show the neurogenic damage (13). Although EAS-EMG was helpful for the diagnosis of MSA (14), its capability in discriminate MSA from other Parkinson's syndromes is uncertain.

Early diagnosis is significant for patients. Currently, biomarkers, imaging markers, and clinical biomarkers are widely applied in the diagnosis of disease. But as mentioned above, discriminating MSA-P from PD based on a single marker is challenging. The combination of multiple indicators may help to distinguish them. About biomarkers, α-syn levels in blood and CSF is easily affected by hemolysis. The collection of blood or CSF is invasive. The saliva acquisition is non-invasive and free of hemolysis contamination. About some examinations, such as Single-Photon Emission Computed Tomography (SPECT) (15), cardiac Metaiodobenzylguanidine scintigraphy (16) are valuable to differentiate MSA from PD. However, these inspections have low sensitivity and specificity, high requirements for equipment and technique, and expensive prices. Although EAS-EMG was moderate invasive, neurogenic damage of EAS-EMG can support the diagnosis of MSA. And it has relatively low technical requirements and low price, which is suitable for clinical promotion.

Therefore, in the present study, we aimed to investigate a new method to make an identification between MSA-P and PD by combining biofluid marker with electrophysiology marker.

## Patients and Methods

### Subjects

This research was approved by the Ethics Committee at Beijing Tiantan Hospital and in accordance with the Declaration of Helsinki. All participants provided the informed consent. This study is an exploratory study. 16 MSA-P and 26 PD patients were enrolled from department of movement disorders disease in Beijing Tiantan Hospital between November 2016 to December 2017. We diagnosed MSA according to the Second consensus criteria (17). We adopted the United Kingdom Parkinson's Disease Society Brain Bank criteria (18) to diagnose PD. Two professional neurologists who expert in neurodegenerative disease confirmed the diagnosis of PD and MSA.

### Datum Collection

Demographic variables (age and sex) and clinical characteristics were collected for each participant. The clinical characteristics included onset age, disease duration, Hoehn-Yahr (H&Y), Unified Parkinson's Disease Rating Scale (UPDRS)-III, and levodopa response. We also evaluated the non-motor symptoms including cognition, mood, and sleep. The cognitive status was assessed using the Mini-Mental State Examination (MMSE) scale and Montreal Cognitive Assessment (MoCA) scale. The emotion assessment adopted Hamilton Anxiety Rating Scale (HAMA) and Hamilton Depression Rating Scale (HAMD). Rapid Eye Movement Sleep Behavior Disorder Screening Questionnaire (RBDSQ) was to evaluate the sleep status. The residual urine ultrasound was to assess the volume of residual urine.

### Measurement of Salivary EVs α-syn

We collected saliva sample from all the participants who were in an unstimulated state. Sample collection time was between 9 a.m. to 11 a.m. After collection, saliva sample was centrifuged in order to clear away particles. The supernatant after centrifugation was reserved at −80°C before using. Then we used XYCQ EV Enrichment Kit to extract EVs from saliva supernatants. Adopting new developed Electrochemiluminescence (ECL) assays which was based on Meso Scale Discovery (MSD) measured the levels of α-syn which included total α-syn (α- syn_Total_), phosphorylated-ser129 α-syn (α-syn_PS129_) and oligomeric α-syn (α-syn_Olig_) in EVs of saliva. The sensitivity of ECL could reach to 0.5 pg/ml (19). Compared with other methods, the ECL has powerful advantages such as high sensitivity and accuracy (20). The detailed methods, including saliva collection and centrifugation, the isolation of salivary EVs, measurement of salivary EVs α-syn, have been reported in our previous study (21).

### External Anal Sphincter Electromyography (EAS-EMG)

The patients were examined with a Nicolet EDX electromyographic evoked potentiometer after admission. Before examination, the patients needed to empty bladder, lie on their left side, and flex hips and knees. The ground wire was placed on the right ankle and the hips were separated to fully expose the perianal area. Iodophor disinfects the perianal skin, and the concentric needle electrode at an acute angle was embedded in the junction of anal skin and mucosa membrane (22), and the depth of the needle was about 0.5~1.0 cm. Using multiple motion unit (MUP) wave acquisition method, at least 20 MUPs were collected, the filter width was 5~10 kHz, the amplifier was 100 μV/div, and the scan speed was 5 ms/div. The time limit measurement was to manually adjust the sweep speed and incorporated the satellite potential. The inspection contents included the average duration, amplitude, phase, satellite potential during light contraction. Satellite potential referred to an additional potential component that appeared before or after the main wave component of the MUP and had an amplitude less than the main wave and a time-locked relationship with the main wave. There is at least 1 ms equipotential line interval between the two (23); satellite potential percentage = (The number of MUPs containing satellite potential/collected total MUPs) × 100%.

### Statistical Analysis

All analyses were performed by SPSS 24.0 (SPSS Inc., Chicago, IL, USA). The Prism 7.0 (GraphPad software, La Jolla, CA, USA) was used to draw graphs. We presented continuous variables with normal distribution as mean ± standard deviation (SD) and the non-normal distribution as median (interquartile range). Two continuous normally distributed variables were analyzed with the independent samples Student's test. The difference of two continuous non-normally distributed was analyzed using Mann-Whitney *U*-test. The chi-square test was used to compare the sex ratio between two groups. We used the logistic regression analysis to make a model. We investigated the diagnostics value of three forms of α-syn, indexes of EAS-EMG, and combined model by using the receiver operator characteristic (ROC) curve. The maximum value of the Youden index corresponds to the cut-off value (Youden index = sensitivity + specificity−1). The *P* value < 0.05 for two-sided was considered as statistically significant.

## Results

### Demographic Characteristics of Participants

The saliva sample were obtained from 16 MSA-P patients (14 probable and 2 possible MSA-P) and 26 PD (14 tremor-predominant, 12 rigidity-predominant). No significant difference in age, sex distribution, disease duration, onset age, UPDRS-III, H&Y was indicated between MSA-P and PD. The improvement rate of UPDRS-III by applying levodopa drugs to evaluate was higher in PD. Demographic and clinical characteristics were shown in Table 1. We also analyzed non-motor symptoms (cognition, mood, sleep) and residual urine between MSA-P and PD in the Supplementary Table 1.

**Table 1:** Demographic and clinical characteristics.

**Characteristics**	**MSA-P (*n* = 16)**	**PD (*n* = 26)**	***P* value**
Sex (Male/Female)	9/7	12/14	0.525
Age (years)	57.31 ± 7.78	56.82 ± 6.45	0.564
Age of onset	54.25 ± 7.68	54.18 ± 6.05	0.465
Disease duration (years)	3.06 ± 1.73	2.64 ± 1.19	0.424
H&Y	2.81 ± 0.75	2.50 ± 0.62	0.067
UPDRS-III	42.75 ± 18.87	40.77 ± 16.00	0.193
Levodopa response (%)	18.25 ± 14.34	40.53 ± 18.92	< 0.0001

*The results expressed as mean ± standard deviation (SD)*.

*MSA-P, multiple system atrophy-parkinsonism; PD, Parkinson's disease; UPDRS-III, Unified Parkinson's Disease Rating Scale-III; H&Y, Hoehn-Yahr*.

### Difference of α-syn Levels in Salivary EVs, Indexes of EAS-EMG Between MSA-P and PD

**Figure 1:**
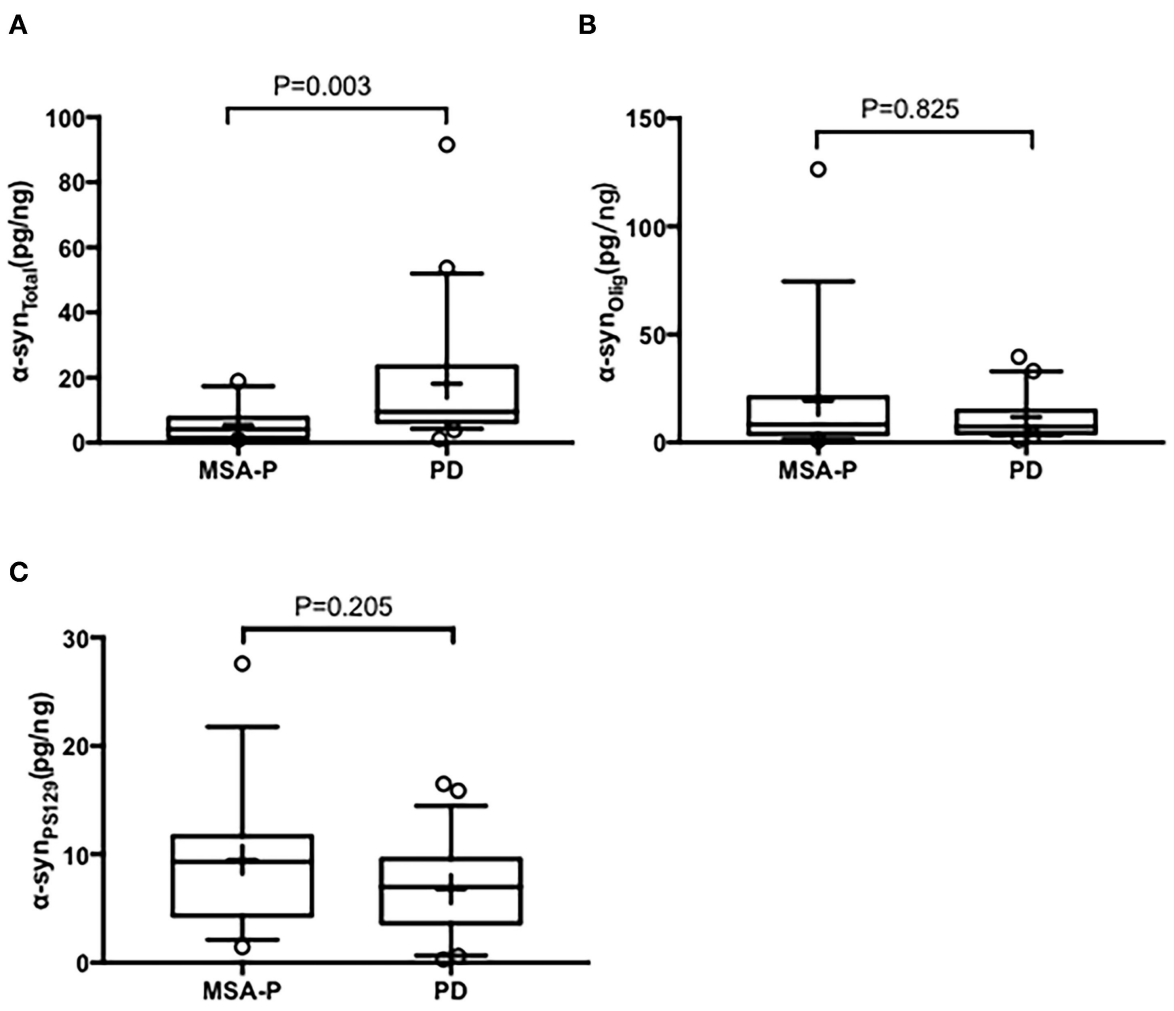
Box plot of total α-syn **(A)**, oligomeric α-syn **(B)**, and phosphorylated-ser129, α-syn **(C)** levels in EVs of saliva between MSA-P and PD. This box plot depicted the median and quartiles. “+” represent the mean value.

**Figure 2:**
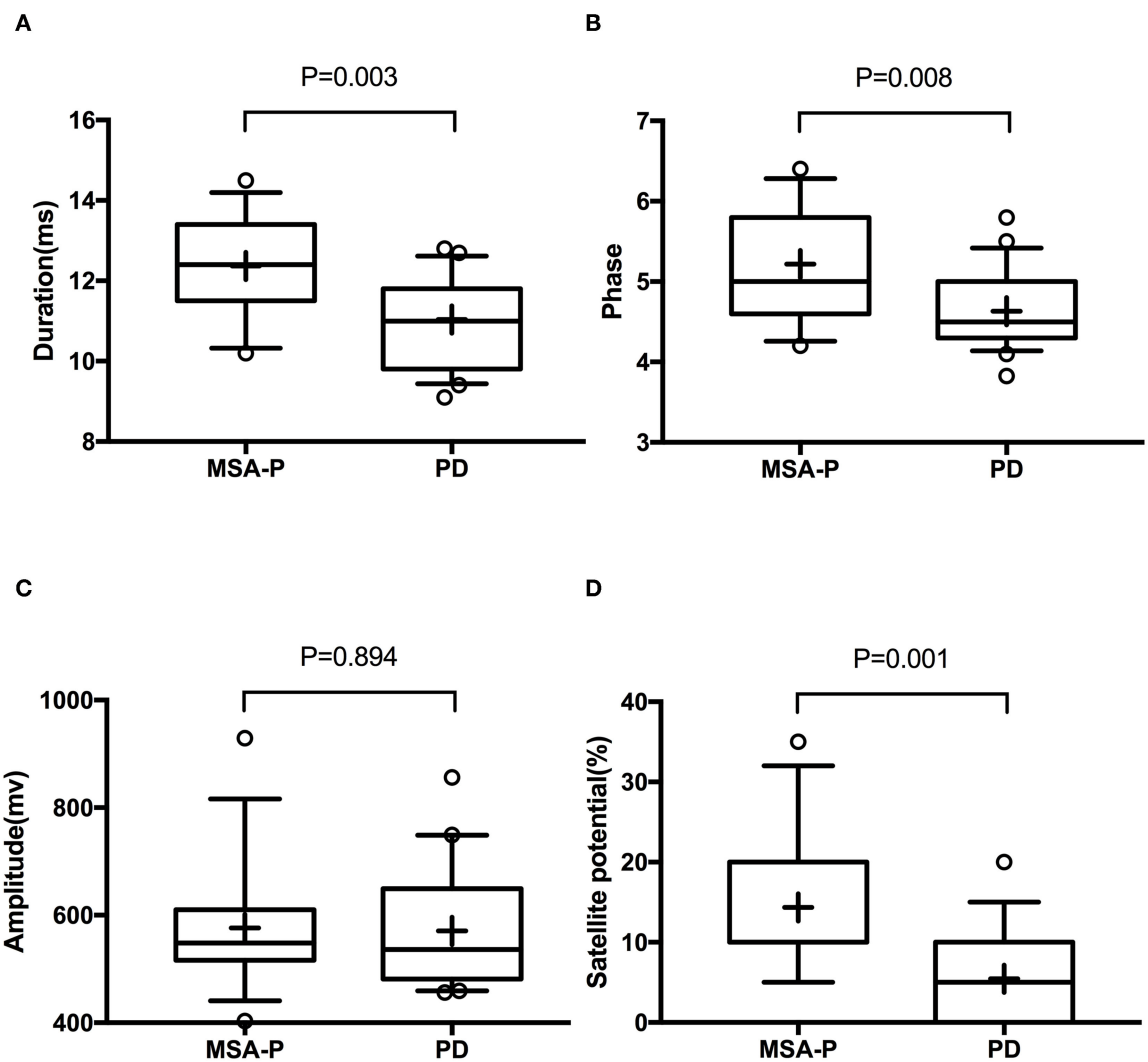
Box plot of duration **(A)**, phase **(B)**, amplitude **(C)**, and satellite potential **(D)** between MSA-P and PD. This box plot depicted the median and quartiles. “+” represent the mean value.

In the EVs of saliva, the α-syn_Total_ levels was lower in MSA-P than PD (*P* = 0.003, Table 2, Figure 1). However, no statistical difference was found in α-syn_Olig_ (*P* = 0.825, Table 2, Figure 1) and α-syn_PS129_ (*P* = 0.205, Table 2, Figure 1); Regarding variables of EAS-EMG, compared with PD, the duration, phase and satellite potential of EAS-EMG in MSA-P were significantly increased (*P* = 0.002, 0.008, 0.001, respectively; Figure 2). There was no statistical difference between two kinds of disease in amplitude (*P* = 0.894; Figure 2).

**Table 2:** The α-syn levels in EVs of saliva, indexes of EAS-EMG between MSA-P and PD.

**Variables**	**MSA-P, *n* = 16**	**PD, *n* = 26**	**Z/t value**	***P* value**
α-syn levels in EVs of saliva, pg/ng				
α-syn_Total_	5.44 (1.50~7.64)	8.07 (4.71~21.65)	−2.868	0.003
α-syn_Olig_	8.25 (3.98~20.99)	7.29 (4.44~14.66)	−0.241	0.825
α-syn_PS129_	9.48 ± 7.06	6.69 ± 3.66	1.307	0.205
Indexes of EAS-EMG				
Duration (ms)	11.90 ± 1.37	10.92 ± 1.10	3.315	0.003
Phase	5.10 ± 0.65	4.63 ± 0.48	3.133	0.008
Amplitude (mv)	590.63 ± 158.21	570.78 ± 108.22	0.135	0.894
Satellite potential (%)	10 (5–20)	5 (0–10)	−3.260	0.001

*The variables described as mean ± standard deviation (SD) and median, interquartile ranges*.

*EVs, extracellular vesicles; EAS-EMG, external anal sphincter electromyography; MSA-P, multiple system atrophy-parkinsonism; PD, Parkinson's disease; α-syn_Total_, total α-synuclein; α-syn_Olig_, oligomeric α-synuclein; α-syn_PS129_, phosphorylatedser129 α-synuclein*.

### Diagnostic Analysis of α-syn_Total_ in Salivary EVs and Indexes of EAS-EMG (Including Duration, Phase and Satellite Potential) Between MSA-P and PD

**Figure 3:**
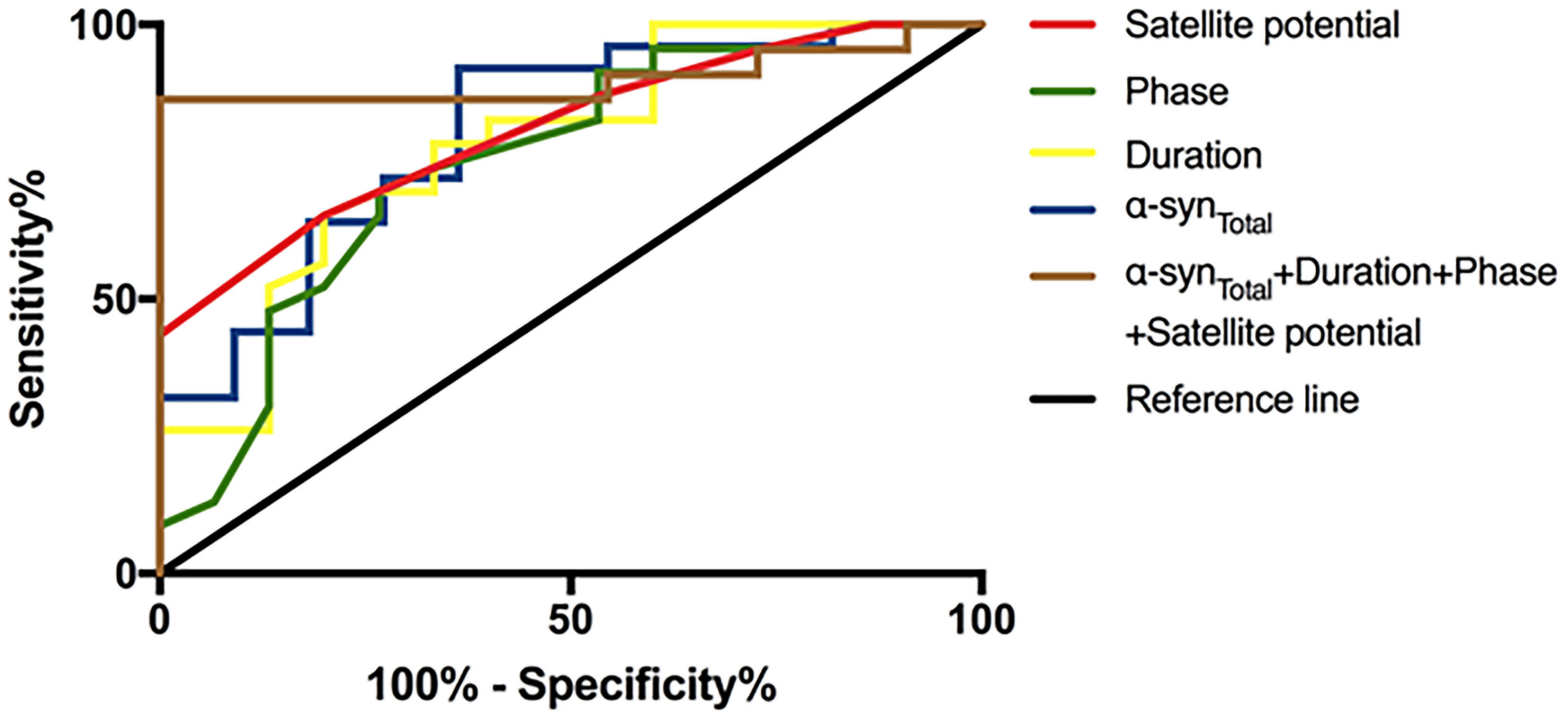
ROC curve of α-syn_Total_ in salivary EVs and indexes of EAS-EMG (including duration, phase and satellite potential) between MSA-P and PD. The red curve, green curve, yellow curve, blue curve, and brown curve represent satellite potential, phase, duration, α-syn_Total_, and integrative model.

To evaluate the utility of α-syn_Total_ levels in EVs of saliva and EAS-EMG in distinguishing MSA-P from PD, ROC curve was performed (Table 3, Figure 3). The α-syn_Total_ 4.46 pg/ng distinguished MSA-P from PD with area under the curve (AUC) 0.804. The sensitivity and specificity were 92% and 64%, respectively. Duration, phase and satellite potential of EAS-EMG discriminated MSA-P from PD with sensitivity of 80% and 73% and 80%, specificity of 65% and 70% and 65%, respectively (duration at a cut-off = 11.4 ms, AUC:0.780; phase at a cut-off = 4.75, AUC:0.751; satellite potential at a cut-off = 7.5%, AUC:0.809). The logistic regression incorporating α-syn_Total_, duration, phase and satellite potential provided a model (−11.038–0.135^*^α-syn_Total_+0.454^*^duration+phase+0.178^*^satellite potential) that increased the utility to diagnose MSA-P. The ROC curve showed that the sensitivity and specificity were 100% and 86% respectively. The AUC value was 0.901 (Table 3, Figure 3).

**Table 3:** ROC curve of α-syn_Total_ in EVs of saliva and indexes of EAS-EMG (including duration, phase, and satellite potential) between MSA-P and PD.

**Variables**	**Cut-off**	**AUC**	**Sensitivity (%)**	**Specificity (%)**	***P* value**	**95% CI**
α-syn_Total_	4.46	0.804	92	64	0.004	0.644–0.963
Duration	11.40	0.780	80	65	0.004	0.627–0.933
Phase	4.75	0.751	73	70	0.010	0.585–0.916
Satellite potential	7.50	0.809	80	65	0.001	0.674–0.943
α-syn_Total_ + Duration + Phase + Satellite potential	NA	0.901	100	86	<0.001	0.789–0.995

*AUC, area under the curve; CI, Confidence Interval; α-syn_Total_, total α-synuclein; NA, not applicable*.

## Discussion

Our study showed that α-syn_Total_ levels in EVs of saliva were lower in MSA-P than PD. However, neither α-syn_Olig_ or α-syn_PS129_ can discriminate MSA-P and PD. Compared with PD, the duration, phase and satellite potential of EAS-EMG in MSA-P were significantly increased. No significant difference was found in amplitude of EAS-EMG between MSA-P and PD patients. The combination of α-syn_Total_ in EVs of saliva and EAS-EMG (including duration, phase and satellite potential) could efficiently distinguish MSA-P from PD. The sensitivity and specificity were 100% and 86%, respectively.

The study about the biomarker of MSA was scarce. This study showed that α-syn_Total_ levels in the EVs of saliva were lower in MSA-P than PD. MSA and PD are both classified as α-synucleinopathy. α-syn can easily aggregated in the extracellular spaces such as cerebrospinal fluid (CSF), serum/plasma, red blood cells (RBCs), and saliva (24). And some studies have made attempts to diagnose MSA using biofluid marker, such as CSF and blood. In CSF, Shi et al. (8) found that although α-syn in CSF were both decreased in PD and MSA patients, but the decrease was more prominent in MSA. In plasma, Lee et al. (6) found that the concentrations of α-syn of MSA patients were relatively low in comparison with PD patients. Compared with CSF and blood, saliva is free of hemolysis (25) and easily available. α-syn in saliva is a more promising biomarker. Our findings suggested that α-syn_Total_ concentrations in EVs of saliva were lower in MSA-P than in PD. The current consequences were also in accordance with previous study. Based on this observation, one possible explanation is that neurodegeneration in MSA is faster or wider than in PD. As might be considered, although no significant difference was found in the disease duration between MSA-P and PD, the clinical stage of MSA-P patients was more advanced than PD patients.

The modified pathological forms of the α-syn can reflect the fundamental neuropathology of α-synucleinopathy more accurately (26). Recent studies (27) have showed that oligomeric α-syn could lead to neuronal cell death because of toxicity. The significant secondary modification about α-syn was phosphorylation. Phosphorylated forms of α-syn can stimulate the progression of fibril (28). And the main type of phosphorylated α-syn which deposited in the LBs was phosphorylated at serine 129 (29). In the current study, we also compared α-syn_Olig_, α-syn_PS129_ levels between MSA-P and PD. It's a pity that no significant difference was shown between two diseases. The previous study also showed that α-syn oligomers/total protein ratio in RBC was not significantly different between PD and MSA. And there was no remarkable difference in oligomeric or phosphorylated α-syn in CSF between PD and MSA. The possible explanation is that method of analysis and small sample size may exert an effect on results.

The neurodegeneration of Onuf's nucleus was the typical pathological feature of MSA (30). The analysis of EAS-EMG which can detects neurogenic damage is significant to identify MSA. Yet there was currently no uniform standard for the neurogenic damage in EAS-EMG (31). It is challenging for doctors to distinguish MSA from other Parkinson's syndromes such as PD. Some study think that EAS-EMG is not helpful for the diagnosis of MSA (32, 33). Currently, the trustworthiness of EAS-EMG is still in hot debate. Consistent with the previous study (34–36), our study also found that the duration, satellite potential, and phase of EAS-EMG in MSA-P were significantly increased compared with PD. There was no statistical difference between two kinds of disease in amplitude. Given the most appropriate parameter to identify MSA is unknown, the ROC curves of duration, phase and satellite potential were analyzed. The satellite potential was the best suitable indicator for the recognition of MSA-P. The result was not fully consistent with the previous research. The possible reasons were the different sample size, and clinical stages of included patients.

Our research showed α-syn_Total_ and EAS-EMG can be applied as complementary diagnostic tool for MSA-P. The sensitivity and specificity of α-syn_Total_ discriminated MSA-P from PD were, respectively, 92% and 64%. The duration, phase and satellite potential of EAS-EMG discriminated MSA-P from PD with sensitivity of 80% and 73% and 80%, specificity of 65% and 70% and 65%, respectively. In order to get the more accurate diagnostic value, we combined α-syn_Total_ with EAS-EMG. The results suggested that the combination of α-syn_Total_ in salivary EVs and EAS-EMG could efficiently diagnose MSA-P with sensitivity of 100% and specificity of 86%. Combining biofluid marker with electrophysiology marker provides a new thought and improves diagnostic accuracy to make an identification between MSA-P and PD. In the further study, we can combine the multiple marker in order to diagnose accurately.

However, our study also has some limitations. Firstly, the sample size of this study is small and we didn't set healthy control. Considering that this study is an exploratory research, we tried to do a preliminary exploration. We will expand the sample size and enroll as many healthy people as possible in the further study. Secondly, different EMG operating systems could also contribute to the different analysis results of EAS-EMG. These technical issues are inevitable. We tried our best to keep consistency in our research populations. Thirdly, the definite diagnosis of these patients was not confirmed according to the pathological results. Nonetheless, all participants were carefully evaluated by professional dyskinesia specialist during hospitalization.

## Conclusions

The combination of α-syn_Total_ levels in EVs of saliva and EAS-EMG (including duration, phase and satellite potential) could efficiently distinguish MSA-P from PD. This study provides a new thought to help the accurate diagnosis of MSA-P. At the same time, the clinical applicability of this finding will be investigated in future studies.

## Data Availability Statement

The raw data supporting the conclusions of this article will be made available by the authors, without undue reservation.

## Ethics Statement

The studies involving human participants were reviewed and approved by the Ethics Committee of Beijing Tiantan Hospital. The patients/participants provided their written informed consent to participate in this study.

## Author Contributions

ZC and YW performed the statistical analysis and drafted this manuscript. YW completed the overall experiment. GL made a revision of manuscript. XW and ZW analyzed the data. TF contributed to the study design. All authors have read and agreed with the final manuscript.

## Conflict of Interest

The authors declare that the research was conducted in the absence of any commercial or financial relationships that could be construed as a potential conflict of interest.
