# Supplementary material for: Differential Diagnosis of Multiple System Atrophy-Parkinsonism and Parkinson's Disease Using α-Synuclein and External Anal Sphincter Electromyography
Source: Front Neurol. 2020 Sep 17;11:1043. doi: 10.3389/fneur.2020.01043 (PMC7527535; doi:10.3389/fneur.2020.01043)
Supplement: Supplementary file 1 [file Table_1.docx]

**Supplementary Table 1:** Clinical evaluations between PD and MSA-P

| Variables | MSA-P(n=16) | PD(n=26) | *P* value |
| --- | --- | --- | --- |
| Residual urine(ml) | 11(2-56） | 1(0-21.5) | 0.066 |
| MMSE | 25.92±4.38 | 25.27±4.84 | 0.663 |
| MoCA | 20.60±4.12 | 21.15±5.24 | 0.675 |
| HAMA | 12.25±7.70 | 13.15±6.51 | 0.764 |
| HAMD | 10.50±8.61 | 12.88±8.05 | 0.789 |
| RBDSQ | 3.57±2.56 | 5.08±3.33 | 0.173 |

The variables described as median, interquartile ranges.

MMSE, Mini-Mental State Examination (MMSE); MoCA, Montreal Cognitive Assessment; HAMA, Hamilton Anxiety Rating Scale; HAMD, Hamilton Depression Rating Scale; RBDSQ, Rapid Eye Movement Sleep Behavior Disorder Screening Questionnaire
